# A Novel Non-Allelic Homologous Recombination Event in a Parent with an 11;22 Reciprocal Translocation Leading to 22q11.2 Deletion Syndrome

**DOI:** 10.3390/genes13091668

**Published:** 2022-09-17

**Authors:** Steven Pastor, Oanh Tran, Daniel E. McGinn, T. Blaine Crowley, Elaine H. Zackai, Donna M. McDonald-McGinn, Beverly S. Emanuel

**Affiliations:** 1 Department of Biomedical and Health Informatics, The Children’s Hospital of Philadelphia, Philadelphia, PA 19104, USA; 2 Division of Human Genetics, The Children’s Hospital of Philadelphia, Philadelphia, PA 19104, USA; 3 Department of Pediatrics, Perelman School of Medicine at the University of Pennsylvania, Philadelphia, PA 19104, USA

**Keywords:** 22q11.2 Deletion Syndrome, microdeletion, optical mapping, recombination, structural variation

## Abstract

The most prevalent microdeletion in the human population occurs at 22q11.2, a region rich in chromosome-specific low copy repeats (LCR22s). The structure of this region has eluded characterization due to a combination of size, regional complexity, and haplotype diversity. To further complicate matters, it is not well represented in the human reference genome. Most individuals with 22q11.2 deletion syndrome (22q11.2DS) carry a de novo, hemizygous deletion approximately 3 Mbp in size occurring by non-allelic homologous recombination (NAHR) mediated by the LCR22s. The ability to fully delineate an individual’s 22q11.2 regional structure will likely be important for studies designed to assess an unaffected individual’s risk for generating rearrangements in germ cells, potentially leading to offspring with 22q11.2DS. Towards understanding these risk factors, optical mapping has been previously employed to successfully elucidate the structure and variation of LCR22s across 30 families affected by 22q11.2DS. The father in one of these families carries a t(11;22)(q23;q11) translocation. Surprisingly, it was determined that he is the parent-of-deletion-origin. NAHR, which occurred between his der(22) and intact chromosome 22, led to a 22q11.2 deletion in his affected child. The unaffected sibling of the proband with 22q11.2DS inherited the father’s normal chromosome 22, which did not aberrantly recombine. This unexpected observation definitively shows that haplotypes that engage in NAHR can also be inherited intact. This study is the first to identify all structures involving a rearranged chromosome 22 that also participates in NAHR leading to a 22q11.2 deletion.

## 1. Introduction

Numerous genetic disorders are associated with rearrangements of chromosome 22, especially deletions and translocations. These include 22q11.2 Deletion Syndrome (22q11.2DS), which is the most common human microdeletion syndrome, and Emanuel Syndrome, which stems from non-disjunction of the derivative chromosome 22 created by the recurrent reciprocal 11;22 translocation [1,2,3]. The complex genomic architecture of several regions of chromosome 22q11 make it vulnerable to the chromosomal changes required to produce these chromosomal disorders.

The presence of multiple AT-rich regions in chromosome 22q11.21 leads to an increased susceptibility for deletions and translocations [4,5]. Specifically, a palindromic AT-rich repeat (PATRR) region is involved in recurrent translocations, including the recurrent reciprocal t(11;22) [3,4,5]. The PATRR-mediated t(11;22) is the most common recurring translocation observed in humans [6]. PATRRs in 11 and 22 make the two regions prone to single-strand breaks which can fuse to form the rearranged chromosomes [7]. This translocation can occur de novo or is often inherited from a translocation carrier. Children of translocation carriers may be carriers themselves, putting them at risk of having a child with Emanuel Syndrome as a result of 3:1 meiotic malsegregation [3]. Interestingly, the translocation has been found to occur in the sperm of normal males [8]. Obviously, studying female oocytes is more difficult, and it is unknown whether the translocation also occurs in oocytes. Individuals with the translocation have between a 1.8 and 5.6% overall risk of producing a child with Emanuel Syndrome, 23–37% increased chance of miscarriage, and ~50% chance for an offspring to inherit the translocation [3].

Chromosome 22q11.2 Deletion Syndrome (22q11.2DS) is the most common chromosomal microdeletion disorder. It occurs in an estimated 1 in every 1000 fetuses [1,9,10]. Affected individuals harbor a hemizygous microdeletion in 22q11.2, approximately 0.7–3 million base pairs in size. About 90–95% of cases result from a de novo non-allelic homologous recombination (NAHR) event in meiosis. The remaining cases inherit a 22q11.2 microdeletion from a parent [1,9,10]. Patients with 22q11.2DS demonstrate wide phenotypic variability including congenital heart disease, craniofacial anomalies, behavioral and learning differences, and an increased risk of developing psychiatric illness, particularly schizophrenia, in adolescence or young adulthood [1].

The chromosome 22q11.2 region contains several large blocks of chromosome 22-specific low copy repeats (LCR22s), making it one of the most structurally complex regions of the genome [11]. This is compounded by the fact that the LCR22s share several highly identical (>95%) segmental duplications [11]. The shared high sequence identity between LCR22s makes them vulnerable to meiotic error [11]. The same characteristics that lead to meiotic error in 22q11.2 LCR22s prevent accurate sequencing and characterization of the region. To date, only fiber fluorescence in situ hybridization (FISH) and Bionano Genomics optical mapping have successfully characterized the extensive variability of LCR22s and have produced contiguous mapped haplotypes [12,13].

Recently, we mapped 30 families, each consisting of probands with 22q11.2DS and their healthy parent(s) [13]. Four individuals were mapped in one of the families: two healthy parents, one proband with 22q11.2DS, and one healthy, unaffected sibling. The father was unique compared to most other parents in this study, as he carries the reciprocal t(11;22)(q23;q11). Interestingly, we found him to be the parent-of-deletion origin for the NAHR event leading to the 22q11.2 deletion in the proband with 22q11.2DS. This was an unexpected result, as prior to this study, we had anticipated that the mother would be the parent-of-deletion-origin. The reason for this assumption is that this particular situation had never been observed in a parent-of-deletion-origin, as no connections between the t(11;22) and the 22q11.2 deletion had previously been identified. Although the reciprocal translocation has never been observed in association with the 22q11.2 deletion, the converse is that since the 22q11.2DS is the most common microdeletion syndrome, it has been observed with other structural abnormalities [14]. For example, 13 patients with 22q11.2DS were also found to have concurrent diagnoses of Severe Combined Immunodeficiency (SCID), Trisomy 8 mosaicism, Bernard-Soulier, CEDNIK syndrome, CHARGE syndrome, cystic fibrosis, 17q12 deletion, G6PD deficiency, von Willebrand, and 1q21.1 syndromes by chance alone [14]. As such, there is a report of incidence in dual diagnoses of 0.9% at one clinical diagnostic center thought to be the result of the high prevalence of 22q11.2DS [14]. Still, despite concurrent diagnoses, no study has ever fully mapped multiple rearrangements in a single parent-of-deletion-origin. Using our optical mapping process, we obtained the whole LCR22 haplotypes of each of the four family members and determined which haplotypes were inherited in the two probands. These data allow us to hypothesize that NAHR leading to the 22q11.2 deletion resulted from a recombination event which occurred within the father’s requisite quadrivalent.

In this study, the optical maps for all individuals of this unique family are elucidated and described. These maps indicate which haplotypes were explicitly involved in NAHR and which structures were deleted and retained after the aberrant recombination. The father who harbors a reciprocal t(11;22) translocation is the parent-of-deletion-origin. Thus, he produced a child with 22q11.2DS when his derivative and normal chromosome 22 participated in NAHR. By comparing maps between family members, we also demonstrate that a haplotype that may participate in NAHR can also be inherited as typically expected.

## 2. Materials and Methods

The four subjects for this study were derived from a previous study of families consisting of two healthy parents and an affected proband with a de novo ~3 Mbp deletion in 22q11.2 recruited through the 22q and You Center at the Children’s Hospital of Philadelphia. The patients and their parents were tested for the presence of the deletion using either a FISH assay with N25 probes (Abbot Molecular, Abbot Park, IL, USA) or the MLPA SALSA P250 DiGeorge diagnostic probe kit (MRC-Holland). All individuals in the study were informed of the research project and provided written consent for their EBV cell lines and DNA to be used for research purposes. The study was approved by the Children’s Hospital of Philadelphia under the Institutional Review Board (IRB) protocol 07-005352.

Eight fluorescently-labeled short tandem repeat polymorphisms (STRPs) or microsatellite markers within the deleted region (Supplemental Table S1) were used to determine the parent-of-deletion-origin. PCR products were amplified using Go Taq (Promega) and analyzed on an ABI 3730 instrument in the Nucleic Acid/Protein core facility at the Children’s Hospital of Philadelphia. The GeneMarker software (https://softgenetics.com/products/genemarker/, Version 2.0, SoftGenetics, State College, PA 16803, USA) was used to analyze the size and relative intensity of each product. A minimum of three informative markers per trio were required to assign parent of origin; five were found for this family. An informative marker would typically consist of the proband with 22q11.2DS matching one parent’s allele, but missing an allele from the other parent.

High Molecular weight DNA isolation was performed based on manufacturer’s protocol (https://bionanogenomics.com/wp-content/uploads/2018/02/30026-Bionano-Prep-Cell-Culture-DNA-Isolation-Protocol.pdf, accessed on 1 June 2020). Briefly, lymphoblast cultures were established for eventual HMW isolation. Lymphoblasts were placed into gel plugs, melted, and purified through drop dialysis. DNA samples with a concentration between 36–150 ng/μL and high viscosity were used in the following DNA labeling experiment.

Purified HMW DNA was labeled according to Bionano Prep Direct Label and Stain (DLS) Protocol (Bionano, #30206, Rev. D). Briefly, HMW DNA was pipetted with labeling master mix and incubated for 2 h in a thermocycler. This mixture was purified with proteinase K and further cleaned on a DLS membrane (supplied by Bionano). Labeled DNA was mixed with staining master mix. Stained, labeled DNA was spun down, homogenized, and stored overnight. Labeled and stained samples with a concentration between 4 and 12 ng/μL were loaded onto a dual flowcell Saphyr chip (Bionano). The chip was covered with a Saphyr clip and placed on the Bionano Saphyr instrument. The Saphyr instrument imaged fluorescently labeled DNA molecules. Generally, after 24–36 h, a flowcell on a Saphyr chip can generate 320–480 Gbp of data.

Tab-separated BNX files containing molecule length, label quality scores, and label locations were output from the Bionano Saphyr optical mapping platform. BNX files were filtered for long ‘length while maintaining the manufacturer’s recommended 320 Gbp of total molecule data. Long molecules were required to uniquely span tandem duplicon modules, some ranging >160 kbp, which is longer than the manufacturer’s default 150 kbp cutoff for molecules. Molecule qualities were assessed by the Bionano Access Molecule Quality Reports and compared to manufacturer’s recommended values (https://bionanogenomics.com/wp-content/uploads/2018/04/30223-Saphyr-Molecule-Quality-Report-Guidelines.pdf, accessed on 1 June 2020). Additional data were collected in samples not meeting these values. After molecule quality filters, de novo assembly of the BNX files was performed using default parameters in Bionano Solve v3.3 and v3.4. Molecules and assembled consensus maps (CMAPs) were aligned to the in silico nicked hg38 reference map. Consensus maps aligned to the hg38 reference map were manually validated by connecting long single DNA molecules across ambiguous segmental duplications, as previously described [12].

Label distribution differences between paralogous 160 kbp mapped elements enabled the chaining of molecules from anchor regions and across tandem 160 kbp elements. As previously defined, 160 kbp modules frequently contained polymorphic labels at the 5′ and 3′ ends [12]. These labels further enabled the segregation of 160 kbp modules within the same haplotype, between haplotypes, and between LCR22A and LCR22D.

Our molecule coverage and data quality cutoffs for CMAP haplotype confirmation were generated as follows. Based on our optical mapping data of independently confirmed genomes, we found that ≥5× coverage of anchor and segmental duplicon-overlapping labels enabled the unambiguous determination of separate haplotypes within a genome and separate duplicons within haplotypes. This cutoff was assuming false positive labeling rates of 3–5% and false negative labeling rates of 9–17%, which were the recommended data quality cutoffs from Bionano Genomics. Genomes with values outside any of the previous ranges were re-run.

Finalized assemblies were manually curated into LCR22A and LCR22D localized haplotypes in the Bionano Access software for visualization (https://bionanogenomics.com/support/software-downloads/, accessed on 1 June 2020). New unique haplotypes were added to the chromosome 22 reference map file used in post-assembly alignments. This alternative reference CMAP catalog was used in subsequent assemblies and alignments for expedient confirmation. All final, curated molecule alignments and NAHR events were imaged from Bionano Access and annotated.

NAHR between LCR22A and LCR22D was determined through the visualization of inherited LCR22 haplotypes in the proband from the parent-of-deletion-origin. Label polymorphisms and label distance discrepancies present in parent-of-deletion-origin haplotype maps compared to proband haplotype maps enabled the reduction of ambiguous recombination sites. LCR22A haplotypes are nearly always heterozygous and label distributions are variable enough to determine exact LCR22A haplotype involved in NAHR. Bionano Access visualization software was used to perform the aforementioned operations by visually comparing maps.

## 3. Results

Using our previously-described optical mapping assembly and haplotype validation approach [12,13], we mapped all 22q11.2 haplotypes in the family. As was done previously, we had to map independent LCR22 regions, since not even the long molecules of optical mapping can connect the unique genetic markers required to link the proximal and distal LCR22s. In other words, if a genome has two maps for LCR22A and two maps for LCR22D, we may not always be able to determine which LCR22A maps are on the same chromosome as the LCR22D maps.

**Figure 1:**
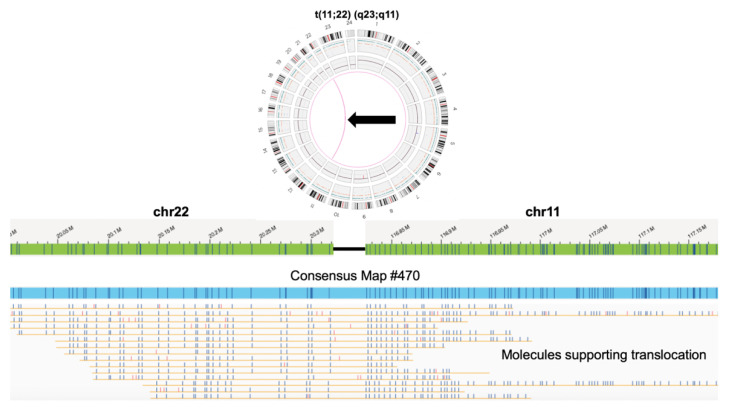
Reciprocal chromosome 11;22 translocation. Top: Bionano optical mapping structural variant caller detected the translocation (red line). Bottom: The same assembled consensus map (#470, blue) mapped to both chromosome 22 (left, green) and chromosome 11 (right, green) hg38 reference maps. The segments of the consensus map that map to chromosome 11 or chromosome 22 do not map to any other loci in the genome. Single DNA molecules (yellow) support the breakpoint of the translocation. This validated that this consensus map was that of the der(22). Blue labels indicate matches and red labels indicated mismatches.

For the father, we first sought to assemble and validate the reciprocal translocation. As indicated in Figure 1, the assembled consensus map with identification number 470 contained an aligned portion mapping to chromosome 22 and chromosome 11. The mapping to 2 chromosomes was validated by independent alignments to only chromosome 11 and only chromosome 22. The breakpoint was identified by comparing DLE-1 labels to the hg38 reference map of either chromosome and was found to be near chr22:20,318,054–20,351,331 and chr11:116,811,929–116,824,680. Single DNA molecules confirmed the translocation breakpoints.

**Figure 2:**
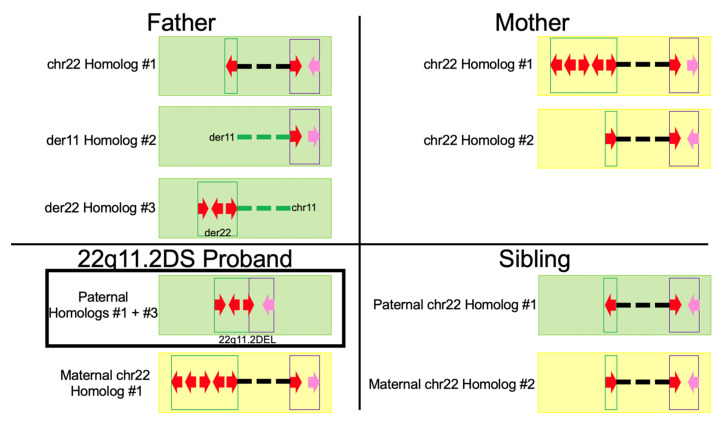
Simplified depiction of the haplotype maps in the family. The father contains 3 homologs of interest; the intact chromosome 22 homolog with no recombination events, the der(11) homolog, and the der(22) homolog. The der(11) and der(22) homologs contain reciprocal translocation breakpoints in LCR22B. The proband contains 2 homologs of interest with the deletion-containing haplotype inherited from the NAHR event in the father. Finally, the mother and unaffected sibling’s haplotypes are shown to the **right**. Green maps indicate inheritance from the father and yellow maps indicate inheritance from the mother. The red arrows indicate 160 kbp modules, whereas the pink arrows indicate the putative LCR22D flanking inversion when facing to the **left.** Dashed lines indicate connection between the respective 22q11.2 region and flanking chromosome 11 regions indicated in the text in the figure. The green dashed lines in the father indicate no inheritance in either offspring and how chromosome 11 from homolog #3 is deleted in the 22q11.2DS proband.

Next, we sought to assemble and validate the independent LCR22A and LCR22D haplotypes in all 4 genomes. These regions were previously found to contain complex copy number variations and orientation changes compared to the reference genome [12,13]. These variations not only occur between unrelated genomes but between haplotypes within genomes. Referring to Figure 2, the father contains 3 homologs of interest; a typical chromosome 22, the der(11) in which 11q23 is connected to distal LCR22B, and the der(22) which contains LCRA and is contiguous until the proximal part of LCR22B which is connected to 11q23. The typical chromosome 22 homolog contains a single, inverted 160 kbp module in LCR22A, a previously-described element with possible influence on NAHR leading to 22q11.2 deletions [12,13,15]. In this same homolog, LCR22D contains a single, reference-orientation 160 kbp module and a previously well-described ~64 kbp inversion from ~21,424,743–21,510,142 [12,13]. The der(11) and der(22) homologs are each connected to chromosome 22 at LCR22B, as described in the translocation breakpoints above. The der(11) homolog contains a single, reference-orientation 160kbp module and no flanking 64 kbp inversion in LCR22D. LCRA of the der(22) homolog contains 3 copies of the 160 kbp module (reference-orientation, inverted, and reference-orientation, from 5′ to 3′).

**Figure 3:**
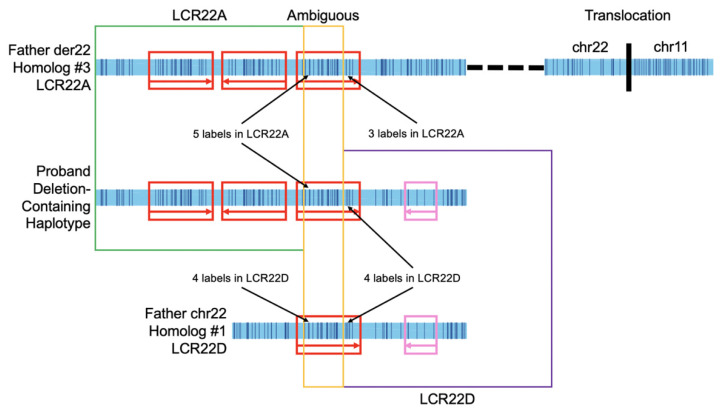
NAHR event in the father leading to 22q11.2 deletion in proband. The 3′-most 160 kbp module in LCR22A of the der(22) homolog #3 from the father recombines with the 160 kbp module in the typical chromosome 22 homolog #1′s LCR22D region. Red boxes indicate 160 kbp modules. The flanking inversion is present in pink in both the father’s LCR22D and the proband’s deletion-containing haplotype, signifying the validated LCR22D portion. LCR22A in the deletion-containing haplotype is confirmed up to the second of the 5′ 5 labels in LCR22A (green box). This is validated, since the father’s LCR22A contains 5 labels and the proband’s haplotype does as well, whereas the father’s LCR22D contains only 4 labels at the same locus. Likewise, LCR22D (purple box) begins where LCR22D contains 4 labels, since that is shared in the proband’s deletion-containing map but not in the father’s LCR22A map, signifying inheritance of the 4 labels from LCR22D. The mustard box signifies ambiguity, as map labels could not determine LCR22A or LCR22D at this ~90 kbp locus. The reciprocal translocation is identified at the right of the der(22) homolog and the dashed line signifies that it is downstream of LCR22A in LCR22B.

The 22q11.2 deletion in the proband was confirmed using specific molecular markers in the MLPA SALSA P250 DiGeorge diagnostic probe kit (MRC-Holland) (see Methods). The deletion-containing haplotype consisted of the three 160 kbp LCRA modules from the father’s der(22) homolog and the 64 kbp inversion in LCRD from the typical chromosome 22 homolog. The NAHR event in the father (described in Figure 3) produced an LCR22A-LCR22D breakpoint within the terminal (i.e., 3′-most) 160 kbp module in LCR22A from the der(22) homolog and the single 160 kbp LCRD module from the typical chromosome 22 homolog. Flanking this 160 kbp module was the inversion often observed in LCR22D. The second chromosome 22 was inherited from the mother. It is her chromosome 22 homolog #1.

The mother and sibling did not have any abnormalities associated with 22q11.2DS or the reciprocal translocation. The mother’s chromosome 22 homolog #1, which was inherited in the proband, contained 5 copies of the 160 kbp module with orientations: inverted, inverted, reference-orientation, inverted, and reference-orientation, from 5′ to 3′ end in LCR22A. This homolog’s LCR22D contained a single, reference-orientation 160 kbp module and no flanking inversion. The second homolog contained a single, reference-orientation 160 kbp module in LCR22A and a single, reference-orientation 160 kbp module with flanking inversion in LCR22D. Finally, the unaffected sibling inherited the typical chromosome 22 homolog from the father (Figure 3; chr22 Homolog #1).

Every assembled haplotype was confirmed with single DNA molecules. Each genome’s assemblies complete with supporting molecules are provided as compressed files.

The father was determined to be the parent-of-deletion-origin with the use of polymorphic molecular markers (see Section 2). This method does not contain the requisite resolution to determine the explicit genetic structures involved in NAHR. To determine these structures, we compared the DLE-1 labels inherited in the affected proband from the father’s LCR22A and LCR22D mapped structures. Label polymorphisms were previously found to exist within paralogous segmental duplicons, especially in the 160 kbp modules [12,13]. These labels enabled the separation of individual 160 kbp copies. As shown in Figure 3, we found the der(22) homolog’s 3′-most LCRA 160 kbp module recombined with the intact chromosome 22′s LCR22D 160 kbp module. In LCR22A, there were 3 labels in the 3′ end of the module, whereas the proband’s deletion-containing haplotype contained 4 labels at this same locus. These 4 labels were also present in the father’s LCR22D 160 kbp module at the same locus. This provided evidence of the NAHR breakpoint having taken place within the LCR22A and LCR22D 160 kbp modules. Further, the inversion present in the LCR22D region of the father’s intact chromosome 22 homolog was present in the proband’s deletion-containing haplotype. This provided additional evidence of the intact chromosome 22 homolog’s involvement in the NAHR event, since the father’s other LCR22D lacked the inversion.

**Figure 4:**
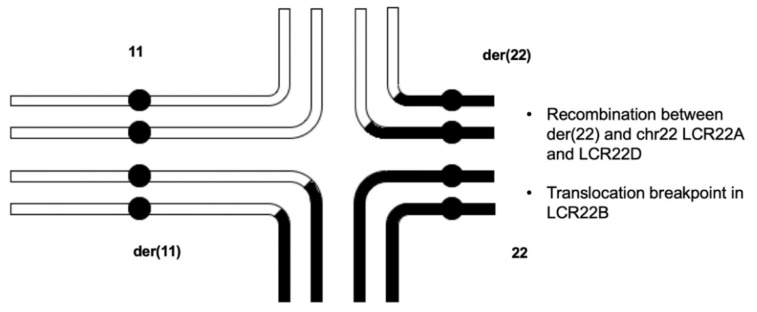
Quadrivalent that undergoes NAHR between the der(22) homolog and the intact chromosome 22 homolog. In meiosis, the homologous regions of the rearranged chromosomes align and recombine in the synaptonemal complex. The aberrant recombination event (NAHR) occurs between the der22 (**top-right**) and the intact chromosome 22 (**bottom-right**). The translocation is not present in the proband’s deletion-containing haplotype, since it is localized within LCR22B, and NAHR took place between LCR22A of the der(22) and LCR22D of the intact chromosome 22.

Before any molecular testing occurred, it was thought that the mother was likely the parent-of-deletion-origin. Because this reciprocal translocation has never been associated with a 22q11.2 deletion, the finding that the father was the parent-of-deletion-origin was unexpected. Thus, the delineation of the mechanism for NAHR became a primary goal, given the presence of the translocation. The quadrivalent for the 11;22 translocation would presumably occur as shown in Figure 4 [16]. Referring to the diagram, the NAHR event was determined to have taken place between the der(22) and intact chromosome 22 homologs. The typical chromosome 11 and the der(11) are presumed to align such that genetic material could recombine between them. The NAHR event, on the other hand, involves the LCR22A region of the der(22) and the LCR22D region of the intact chromosome 22. Because the translocation occurs in LCR22B, the translocated portion of chr11 on the der(22) is not included in the deleted chr22. The deleted recombinant chromosome segregated into the gamete that provided the 22q11.2DS proband’s deletion-containing haplotype.

## 4. Discussion

Our previous work on 30 families with 22q11.2DS probands and healthy parents generated bioinformatics methods to validate the complex structures in the 22q11.2 region [13]. Using these well-defined methods, we assembled and validated complete LCR22 haplotypes in 4 related individuals. The family consists of a father who carries the t(11;22)(q23;q11) rearrangement, a mother with neither 22q11.2DS nor the reciprocal translocation, a proband with 22q11.2DS, and an unaffected sibling. Polymorphic DLE-1 labels clearly defined the reciprocal translocation and its connection to specific LCR22A and LCR22D structures in the father. The inheritance of polymorphic DLE1 labels enabled the analysis and validation of the NAHR event in the 22q11.2DS proband.

In our previous studies, unprecedented variabilities in copy number and orientations of elements in LCR22A and LCR22D were observed [12,13]. This was especially pronounced in the 160 kbp modules. Indeed, upon assembling the haplotypes in the family of 4 in this study, we found similar variability in that there were 4 unique LCR22A haplotypes between the parents. Both parents were heterozygous for LCR22A segmental duplication structures, a common feature found when mapping the LCR22A region across individuals [13]. Although this variability makes sequencing challenging and increases the difficulty of assembling and validating the haplotypes using optical mapping, it allows us to determine the structures surrounding the 22q11.2 breakpoints and directly observe the position of NAHR events in parents-of-deletion-origin. Indeed, both the unique haplotype structures determined from copy number variation and orientation differences of the 160 kbp modules and the polymorphic DLE-1 labels within and between paralogous 160 kbp modules enabled the validation of the aforementioned processes.

The highly identical segmental duplications and extreme variability likely play a role in the instability of chromosome 22, leading to frequent translocations, deletions, duplications, and polymorphisms. In LCR22B, a palindromic AT-rich repeat lies at the junction of the chromosome 11;22 reciprocal translocation. This translocation results in a der(11), connected to the distal end of LCR22B of chromosome 22 and continues to the end of the q arm of chromosome 22, and a der(22) connected at the proximal portion of LCR22B to the distal end of chromosome 11. Despite this, this recurrent reciprocal translocation had yet to be observed in association with a NAHR event leading to a ~3 Mbp hemizygous 22q11.2 deletion. Carriers of the translocation may produce offspring affected by Emanuel Syndrome, which is often how the translocation is identified.

The translocation can create errors with segregation of the involved chromosomes, most notably the der(22). This often leads to difficulty in conception and multiple miscarriages but has not been directly connected to NAHR events leading to 22q11.2 deletions. In this study, we have clearly identified a case where the der(22) homolog participated in NAHR, which instead generated a 22q11.2 deletion. Thus, in this male translocation carrier, a NAHR event close to the centromere of his chromosome 22 homologs precluded an “exchangeless” configuration. Such events might lead to non-disjunction of the der(22) with the normal 22, which is the more typical abnormal meiotic result of this t(11;22) rearrangement. Although the study that demonstrated the finding that absence of crossovers seem to prevail in chromosome 21s that undergo non-disjunction was performed in oocytes [17], it might also be translatable to male meiosis. It is important to note that due to the relative rarity of 22q11.2DS, it may be unusual to find additional cases where the parent-of-deletion-origin is a reciprocal translocation carrier, but this case should alert geneticists to the potential risk of 22q11.2DS in offspring of carriers, in addition to the risk for 3:1 meiotic malsegregation leading to Emanuel Syndrome.

Using optical mapping, we were able to identify the reciprocal translocation in a father of a family with two offspring and found the translocation breakpoint to be the characteristic t(11;22) rearrangement occurring within the PATRRs on chromosomes 11 and 22. To our surprise, this individual was also determined to be the parent-of-deletion-origin leading to a hemizygous 22q11.2 deletion in a proband. Our previous work has identified the reference-orientation 160 kbp module as the predominant structure where NAHR occurs between LCR22A and LCR22D. It was not surprising to see that this was also the case for this particular deletion. An additional surprise, however, was that the unaffected sibling inherited the typical chromosome 22 from the father. This same homolog participated in NAHR providing the LCR22D segment of the deletion-containing haplotype in the affected proband. Thus, the160 kbp modules which recombine to form ~3 Mbp hemizygous deletions may also be inherited in the typical fashion. This has important implications for the study of whether there are predisposing structures leading to NAHR creating 22q11.2 deletions. It indicates that even if a parent harbors the frequently-recombining structures, NAHR may not occur. It is likely that although these structures are frequently NAHR participants, they are not 100% penetrant. Indeed, evidence in our previous study of 30 families supports this finding, since several haplotypes did not participate in NAHR that were identical to haplotypes which did [13]. This is not surprising, as there are few families with multiple 22q11.2DS offspring in the absence of an affected parent [1] Future studies of families with healthy parents, one affected proband, and unaffected siblings will likely generate useful frequencies and inferences related to this potential configurational risk factor.

## 5. Conclusions

The 22q11.2DS is the most common microdeletion syndrome and has been paired with other structural abnormalities [14]. Nonetheless, as previously mentioned, the reciprocal translocation has never been observed in association with the 22q11.2DS. This work demonstrates the finding of a structural 22q anomaly co-occurring with 22q11.2DS and suggests that even if one has a balanced translocation, they can still produce a 22q11.2 deletion. Thus, in this study, we have successfully mapped and validated a recurrent reciprocal translocation associated with NAHR leading to a hemizygous 22q11.2 deletion. The maps indicate the chromosomal structures involved in the NAHR event and the products are fully elucidated.

## Data Availability

Cell lines used to map repeats are available upon request. Assembled genome map data and raw molecules have been deposited to the NCBI BioProject database (https://www.ncbi.nlm.nih.gov/bioproject/) under the accession: PRJNA640411. The custom scripts used in the study are available from https://github.com/stevenpastor/nahr-22q.

## Supplementary Materials

The following supporting information can be downloaded at: https://www.mdpi.com/article/10.3390/genes13091668/s1, Table S1: Parent-of-Deletion-Origin Markers.
